# Towards a Virtual Fencing System: Training Domestic Sheep Using Audio Stimuli

**DOI:** 10.3390/ani12212920

**Published:** 2022-10-25

**Authors:** Natasa Kleanthous, Abir Hussain, Jennifer Sneddon, Wasiq Khan, Bilal Khan, Zeyar Aung, Panos Liatsis

**Affiliations:** 1 Department of Electrical Engineering and Computer Science, Khalifa University of Science and Technology, Abu Dhabi P.O. Box 127788, United Arab Emirates; 2 Department of Electrical Engineering, University of Sharjah, Sharjah P.O. Box 27272, United Arab Emirates; 3 Faculty of Engineering and Technology, Liverpool John Moores University, Liverpool L3 3AF, UK; 4 School of Biological and Environmental Sciences, Liverpool John Moores University, Liverpool L3 3AF, UK; 5 School of Computer Science and Engineering, California State University San Bernardino, 5500 University Parkway, San Bernardino, CA 92407, USA

**Keywords:** animal behaviour, audio stimuli, sheep response, virtual fence

## Abstract

**Simple Summary:**

Virtual fencing is useful for restricting animal movement in a controlled environment with cost and maintenance benefits over physical fencing. Physical fencing is also rigid, which does not allow for flexible use of grazing resources by farmers. The proposed study describes the use of a welfare-friendly virtual fence system over a typically used electric shock fence as an additional stimulus to restrict animal access to designated areas. The virtual fence was operated by an acoustic device, carried on the individual sheep. Sounds in the range of 125 Hz to 17 kHz and white noise were used to discourage seven Hebridean ewes from approaching a restricted area and feeding at a feed bowl. Two trials, performed a year apart, revealed that sounds in the range 125 to 440 Hz; 10 to 17 KHz could prevent sheep from approaching a feeding station (success rate 90%). In 78.5% of the trials, the animal behavior was observed to be the undisturbed movement away from the feed bowl when the acoustic stimulus was employed.

**Abstract:**

Fencing in livestock management is essential for location and movement control yet with conventional methods to require close labour supervision, leading to increased costs and reduced flexibility. Consequently, virtual fencing systems (VF) have recently gained noticeable attention as an effective method for the maintenance and control of restricted areas for animals. Existing systems to control animal movement use audio followed by controversial electric shocks which are prohibited in various countries. Accordingly, the present work has investigated the sole application of audio signals in training and managing animal behaviour. Audio cues in the range of 125–17 kHz were used to prohibit the entrance of seven Hebridean ewes from a restricted area with a feed bowl. Two trials were performed over the period of a year which were video recorded. Sound signals were activated when the animal approached a feed bowl and a restricted area with no feed bowl present. Results from both trials demonstrated that white noise and sounds in the frequency ranges of 125–440 Hz to 10–17 kHz successfully discouraged animals from entering a specific area with an overall success rate of 89.88% (white noise: 92.28%, 10–14 kHz: 89.13%, 15–17 kHz: 88.48%, 125–440 Hz: 88.44%). The study demonstrated that unaided audio stimuli were effective at managing virtual fencing for sheep.

## 1. Introduction

A Virtual Fencing (VF) system is a computerized method with inherent features to create spatial boundaries of custom geometric size and shape without any physical fences or barriers. The study and use of VF (or Fenceless) systems have gained momentum over the past three to four decades due to their salient advantages over traditional systems in terms of the flexibility (i.e., customized virtual zones of animal monitoring), reduced cost, and lower maintenance demand [1,2]. Although physical fences are designed with 100% stock-proof capabilities, expenditure on agricultural fencing in grazed farmlands has been among the most expensive infrastructure cost in the 19th century [3,4]. Cost reduction via new effective alternatives for livestock containment has therefore been of significant undertaking. Consequently, VF is considered by the agricultural community as the next generation of physical fences which can significantly reduce the construction/existence of physical barriers and cut the associated costs. Features that can be proven useful for replacing physical barriers for control animal movement and position in grazing systems include visual, auditory, and possibly olfactory cues [5]. A VF system with a modularized toolbox of the above features (but not limited to) could provide information about the animal’s location and changes in grazing intensity on pasture resources in real time [6]. With the help of such evidential information, monitoring animal spatial distribution may lead to improved decision making in regards to efficient land utilization [7], to help prevent soil erosion and soil and water contamination [8]. VF systems can also be used to train animals for a smart response to such stimuli by manipulating real time information from these cues [5,9]. In this regard, VF can be utilized for domestic sheep (*ovis aries*) that are reported to have excellent learning capability and memory compared to other farm animals [10].

Recent literature on animal behaviour reports various studies that investigated the abilities of cattle and sheep to learn and respond to a virtual boundary based on cues associated with negative consequences [11,12,13,14]. For example, a sound warning should be emitted when the animal approaches a restricted area. If the animal continues forward, the subsequent action should be an electric shock. Employing an acoustic approach can prove to be useful for the animals to learn to associate electric shocks with sounds and to prevent them from proceeding further upon the emission of the sound cue. Such a learning approach with the use of audio signals with the stressor of the electric shock can allow the animal to adapt to the negative consequences. It has been reported that training animals using an electric stimulus could have a negative impact on their welfare [15,16], and this has led to their use on sheep to become illegal, as is the case in the UK [17].

Research and development on the ability of sheep to perform a specific task in response to an auditory stimulus alone has significantly been lacking. Morris et al. [9] proposed the use of auditory and visual cues to study the ability of sheep to complete an expected task. The study performed by Morris et al. used 20 merino ewes, where the sheep population was split into two halves with visual and auditory cues, respectively to perform the experiments. A continuous audio cue of 392 Hz originated from speakers, positioned above the feed buckets of the sheep. The study reported that sheep could not learn to respond to the audio cues since the learning probability did not increase over the testing period. The authors suggested a potential for sheep learning from audio cues and the need for further research [9]. Directional stimuli can be used to move animals in a desired direction as this could have commercial applications.

Heffner [18] generated data on frequencies that could be heard by several animal species including humans. The study observed that sheep are able to hear in the range between 125 Hz and 42 kHz, with 10 kHz identified as the most sensitive hearing frequency. Moreover, further study and investigation on the intensity and frequency of sounds were proposed to train animals. In [19,20], it was reported that using intermittent sound pulses as opposed to a continuous stimulus may prove more appropriate since discontinuous sound are effective in producing better response. Umstatter et al. [21] proposed an approach of controlling cattle location via broadcast audio preferred over electric stimuli. The experiment was based on 38 animals with loudspeakers placed around a small paddock. The loudspeakers played unpleasant sounds in the ranges of 8 kHz and 8–10 kHz, and one acute sound. Movement sensors were located closely and linked to the loudspeakers, triggering sounds when the animals approached the restricted area. Although, as per the study, unpleasant and acute sounds can control cattle location, the approach was ineffective in replacing conventional fences with a proposal for further research. 

According to the aforementioned studies, training animals to respond and learn virtual fences based on various sources of stimulation is a feasible yet challenging task. Despite mounting literature in the area, further investment in terms of the research and development is desirable for the use of only acoustic sounds, especially in the case of sheep. Therefore, the present study aims to fill the gap and test the feasibility of removing the use of electric shocks and, instead, manage animal behaviour with audio signals alone, thus leading to the development of a welfare-friendly virtual fence system. In our experiment, the hypothesis was set to be a range of audio cues to generate an aversive stimulus to restrict the animals in a virtual fence. To the best of our knowledge, this is the first study that analyses the effect of various sound frequencies and white noise without pairing an electric stimulus as a method to impose a penalty mechanism for sheep. 

The experiments performed in this study were targeted to address various outstanding questions with respect to: (i) restricting sheep and enforcing a change of course via only the acoustic cues without the need for a stressor; (ii) the identification of the most effective audio frequencies in restricting sheep access to an attractant or a restricted area; (iii) assessing robust correlations between the time to respond to the sound with various factors (e.g., the attractant, animal personality, and audio frequency), and (iv) identifying the existence of a correlation between sheep personality types with the latency of the animal to respond to the emitted sounds. 

## 2. Materials and Methods

### 2.1. Ethical Statement, Animals, and Location

The Senior Research Officer and LSSU Manager of Liverpool John Moores University approved the experimental protocol (approval AH_NKO/2018-13). The two trials were conducted in Shotwick Village near Chester, UK (333792, 371848 • Lat/Long). Seven Hebridean ewes, aged 5–14 years, were used. Throughout the experiments, the animals were free to use the entire area of the paddock and have ad libitum access to grass and water.

Rare breeds of sheep are reported to be significantly more averse to experimental deterrents than heritage or commercial breeds (e.g., withdrawal on approach of a human with a device for administering a paint mark [22] or the presence of a dog outside the pen [15,23]. Hebridean sheep are classified as a heritage breed and are often used in conservation grazing management schemes in the UK. Hebridean sheep are therefore comparable to commercial breeds in terms of grazing management requirements that might benefit from acoustic fencing. Isolation is extremely stressful for any breed of sheep [11,23] and provides an unacceptable source of variation in response to experimental treatments [23]. This is because the emotional state of a sheep affects its concentration, decision making powers and memory [24]. Visual cues from flock members are of paramount importance in influencing behaviour via position and body language [19].

### 2.2. Equipment for Delivering External Stimuli

A commercial Bluetooth speaker (EWA A106 Pro Wireless Mini Bluetooth Speaker) was attached to the animal’s collar using a small case. The speaker weighted 176 grams, with dimensions of 4.8 × 4.8 × 3.84 cm. Additionally, to test animal response to audio signals, a custom sound system was developed in the Cycling ‘74 MAX/MSP’ visual programming language. The system was paired with the collar to manually send audio cues to the animal for testing. It was able to generate white noise (noise that contains a mixture of all audible frequencies that human ears can hear (about 20 hertz to 20 kilohertz. This type of noise includes low-, midrange- and high-frequency sounds) and sounds in the range of 100–20 kHz, which lie within the sheep’s hearing range [18]. The mode of the sound could be set as either intermittent or continuous, while the volume was manually adjusted. Once a sound was emitted, a log file was created, reporting the start time of the sound, the type of the sound (e.g., sine wave, white noise), the volume level, and stop time. To video record the behaviour of the animals, a Canon SX720HS camera (Canon Europa N.V, Bovenkerkerweg 59, 1185 XB Amstelveen, The Netherlands) was used. At the end of the experiments, the video recordings were time synchronized with the log files extracted from the sound system to mark the reaction of the animals based on each sound.

### 2.3. Experimental Protocol

#### 2.3.1. Overview of the Experimental Setting

A small group of seven Hebridean ewes was introduced in the study, with four bold and three shy in personality, in terms of the reaction time and magnitude of their response to an audio cue in the presence of a feed attractant, with age range of 4–12 years. Sheep behaviour is remarkably consistent over time, thus using a small stable group for repeated studies is considered appropriate [17]. The ewes were kept in two contiguous paddocks of 20 × 60 m, making up an area of ¾ acre in total. The experiments took place in one of these paddocks. The acoustic stimuli were applied to the sheep on an individual basis, while they remained in their flock, using feed of high calorific value (e.g., sugar beet shreds) as a strong attractant, since the strength of attraction to feed bowl in experiments involving small groups of sheep has been positively correlated with its caloric value [22]. In the experimental setting, personality (i.e., bold, shy) was not set to influence the number of stimuli sheep were subjected to. 

A challenge when designing the experiments is to reduce the use of animals in the research. We followed the approach outlined by Kortzfleisch et al. [25] in our experimental design. We used a stable social group and repeated observations randomly on each individual over two time periods of two to three weeks long, a year apart. Habituation could, therefore, be examined within and between the two trials as well as personality types via response to novel stimuli and to humans. Sheep were classified as bold and shy based on their approach to novel stimuli or people.

In the experiments, aiming to eliminate unethical levels of stress on sheep and sources of uncontrolled variation in the behavioural data, the experiments with an acoustic deterrent were performed in the normal small flock situation. The human observers were people that the flock were very familiar with. The space available for a given reaction to an auditory stimulus was ample, so that any stress associated with a sensation of undue confinement would not affect behavioural responses. This flock-level testing protocol was also designed to emulate a commercial setting, where several sheep would be competing for an attractive feed resource. This competitive situation created the strongest drive for a given sheep to ignore any acoustic stimuli. The impact of a sheep’s reaction to acoustic stimuli on other members of the flock could also be assessed in terms of how efficient the system was at influencing more than one animal. Only one animal, therefore, wore the collar emitting the acoustic stimulus at any given time. 

#### 2.3.2. Acoustic Treatments and Response Variable

The experimental procedure was common to both trials, described as follows. The ewes were penned and one ewe was randomly selected and fitted with the collar. The collar was easy to fasten and unfasten to minimize the time an animal had to be restrained. The animals had approximately 20–30 min to settle down before each experimental session and were released at the same time. At the end of each experimental session, the animal wearing the collar was returned to the pen to have its collar removed, and then it was fitted to the next animal. Once testing was completed on all animals, the animals were released in their normal area of pasture. 

The video recordings were time synchronized with the log file generated from the sound system to label the response on each sound. The sounds used were in the range of 125–17 kHz, in addition to white noise with random volume levels between 25% and 100%. The frequency type was divided into six groups within the ranges as follows: group 1: low-level starting from 125–440 Hz, group 2: 1–5 kHz, group 3: 6–9 kHz, group 4: 10–14 kHz, group 5: 15–17 kHz, and group 6: white noise with a mixture of frequencies. These acoustic stimuli were randomly applied (starting from the minimum frequency (125 Hz) of the group increasing to the highest (17 kHz) to the sheep with the collar, in series, for indicative amounts of time up to 15 s, until an expected behaviour was observed. The time period to switch off the sound was set to 15 s when no response to a stimulus was observed. The immediate pause of the sound was considered as a reward for the animal which was labelled as favourable behaviour. Mutually exclusive behavioural responses of the animals exposed to the acoustic stimuli were categorized as: (1) turn and walk away; (2) turn and run; (3) stop; and (4) no response (i.e., the animal continued to walk towards the restricted area, or when the animal responded by further moving forward).

### 2.4. Trial 1: Exploring the Effect of Frequency Bands on Animal Response 

**Figure 1:**
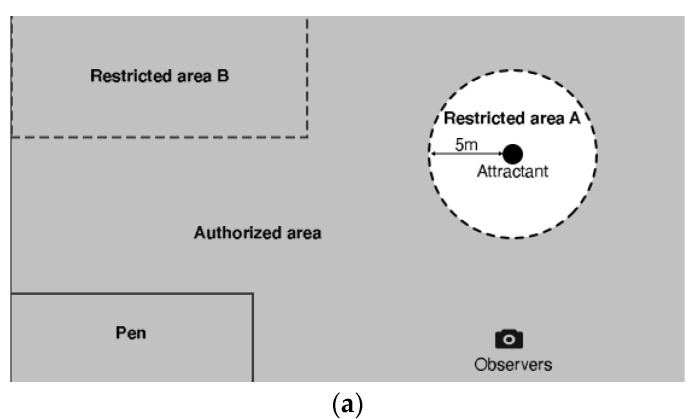

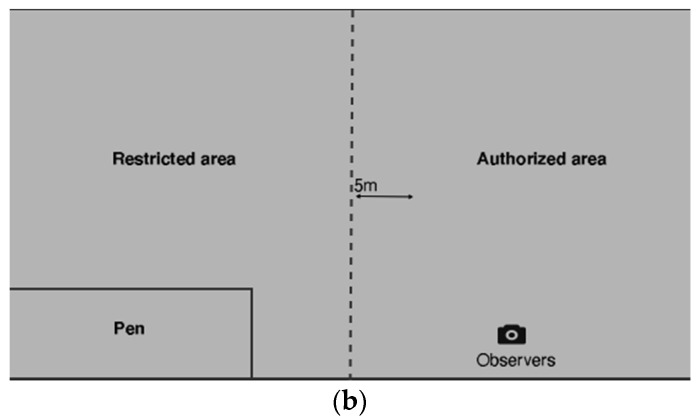
Experimental settings, where both layouts were surrounded by physical fences. (**a**) Experimental layout used in both experiments. The attractant was a bowl filled with. A sound was emitted as soon as the animals were 5 m away from the bowl. (**b**) Experimental layout only used in experiment 2. The pasture was divided into two areas (authorized and restricted). The dashed line represents the end of the authorized area. The sound was emitted as soon as the animals were within 5 m of the dashed line.

The first trial was conducted in August 2018 (12 days in total, 6 rounds of measurement studies, each 2 days apart from the previous) with the layout as shown in Figure 1a. The hypothesis was set to determine the most effective frequencies for restricting animal access to a feed bowl with sugar beet shreds and quantify animal response time (in seconds). Two operators conducted the experiments where operators 1 and 2 were assigned the tasks to observe the animal and send acoustic signals through the customized sound system to the Bluetooth speaker attached to the collar on the sheep, respectively. To ensure consistency, stone markings were placed at 5 m around the feed bowl to accurately determine the precise sound emission time and provide sufficient time for the sheep to respond. The instruction for the sound emission device operator would be communicated via the operator in charge of the video recordings. As shown in Figure 1a, the animals could use the whole pasture with the exception of the area of the pen, and the pasture surrounding the feed bowl.

During this experiment, six sound frequencies were applied as the acoustic stimulus as follows; (1) white noise (mixed frequency); (2) 125–440 Hz; (3) 1–5 kHz; (4) 6–9 kHz; (5) 10–14 kHz; and (6) 15–17 kHz. The behavioural response was categorized as: (1) turn and walk away; (2) turn and run; (3) stop; or (4) no response (acoustic stimulus had no influence). The animal wearing the collar was encouraged to approach the feed and then an intermittent sound was emitted for a maximum of 15 s in case no response was observed. Intermittent white noise and sine wave sounds were also used since discontinuous noise has been proven to generate better responses in animals [19,20]. Four volume levels (25%, 50%, 75%, 100%) were used, ranging from the dB level at 0.0 to −3.0 full scale. Volume levels per experiment in this trial were randomly selected.

### 2.5. Trial 2: Exploring the Effect of Sound Duration on Animal Response 

The second trial was conducted in July 2019 with the same configuration as the first trial. The focus of this trial was to identify: (i) robust correlations of the sheep response time with various factors (the attractant, animal personality, frequency); and (ii) the correlation of the sheep personality type and their response time. In this trial, only frequency bands generating a noticeable animal response (i.e., a positive response rate over 70%) in the first trial were chosen (refer to the Results section Table 1). The location setup with the same configuration as in first trial was first chosen, followed by the modified setup as shown in Figure 1b, with no feed bowl. Sounds were then emitted in these configurations when the animal was standing or walking towards the restricted area. Upon the animal’s range of 5 m from the restricted area, an audio signal was emitted for a maximum of 15 s which was ceased in the following cases—turn and walk away, turn and run, stop. 

## 3. Statistical Analysis

IBM SPSS Statistics (software version 26, creator IBM, country: New York, United States) was used for data analysis that included “duration” as the dependent variable, which was tested against “sheep_type”, “frequency”, “attractant”, and “response”. The analysis was conducted to interrogate the effect of sheep personality type and the applied audio stimulus on the duration of response. A generalized linear model (GLM) [26] under the Tweedie distribution [27] with the Log Link function was used that utilizes a training method for various sets of regression models. GLM attempts to represent the relationship between the independent variables X and the dependent variable Y through an approximate additive/linear mapping that can be useful when such a definitive additive or linear relationship is missing. GLM consists of a linear predictor (η_ι_), a link function (g), and a variance function var (Y_i_) as shown in Equations (1), (2), and (3), respectively:

η_i_ = β_0_ + β_1_x_1i_ +…+ β_ρ_x_ρi,_ (1)

where the β’s are the coefficients to be estimated, for i = 1,…,ρ, and ρ is the number of independent variables. The link function is given by:

g(μ_i_) = η_ι,_ (2)

and describes the dependence of the mean μi on the linear predictor. Finally, the variance function,

Var(Y_i_) = Φvar(μ_i_), (3)

describes the variance on the mean, where Φ is the dispersion parameter. Further information on the generalized linear model can be found in [26,28].

The main effects and interactions of frequency, attractant, sheep personality type and response on the duration were analysed using the Wald chi-square test [29], where *p* < 0.05 was considered to be statistically significant. The results provided information on whether there is a significant effect between the time to respond to the sound and (1) the attractant; (2) personality type of the animal (3) response; and (4) frequency. The analyses illustrated whether faster or stronger responses were obtained from animals exposed to any of the acoustic stimuli. The data obtained are expected to contribute to future designs of audio-based VF systems.

## 4. Results

Table 1 presents the number of repetitions using each band and the number and type of responses. From Table 1, it can be observed that the frequency range of 1–9 kHz failed to attain a substantial response from the animals, since the success rate was relatively modest (i.e., 47.37% for 1–5 kHz, and 35.29% for 6–9 kHz) and, thus, these two bands were excluded from trial 2. 

From the Table 1, it can be observed that white noise was 100% successful in restricting animal access to the feed bowl, followed by the 15–17 kHz frequency range with an 84.21% response. The frequency bands of 125–440 Hz and 10–14 kHz restricted animal access with success rates of 74.29% and 78.95%, respectively. The highest score where the animals turned and walk away from the feed bowl was achieved using frequencies in the range of 15–17 kHz, in contrast to white noise, where the animals turned and walked away only 42.31% of the time. When emitting sounds in the frequency bands of 125–440 Hz and 10–14 kHz, the animals turned and walked away from the bowl with rates of 69.23% and 66.67%, respectively. Animals turned and ran away from the feed bowl 48.08% of the time when white noise was emitted, contrary to the frequency band of 15–17 kHz, where the animals did not run.

### 4.1. Testing the Selected Sounds vs. Category of Animal Response

The results from the second trial are presented in Table 2. During the trial, an average response rate of 90.62% was achieved. The highest response for a specific type of audio stimulus was 92.86%, achieved using low frequency sounds between 125 and 440 Hz. The lowest response rate, i.e., 89.27%, was recorded when using audio signals in the 15–17 kHz range. The average rate of response for animals turning and walking away was 82.26%, with the highest rate of this response (90.16%) recorded at the 15–17 kHz range. With regard to the behaviour where animals turned and ran, this occurred 12.83% of the time when white noise was emitted. The animals did not run when the sound frequency was between 15 and 17 kHz. Based on all selected frequency sounds, the animals stopped while walking towards the bowl between 9.84% and 14.93% of the time. When considering all selected sounds, only 9.38% of the time did the animals not respond to the stimulus and instead proceeded towards the bowl.

### 4.2. Duration Statistics and Effect of Sheep Personality Type, Attractant, Frequency, and Response on Duration 

The minimum, maximum, mean, and standard deviation of the dependent variable (stimulus duration) are shown in Table 3. Table 4 provides information on the results from the overall GLM model [28]. The omnibus test is a likelihood-ratio chi-square test of the model versus the null hypothesis [30]. A significance value of less than 0.05 indicates that the current model is more likely than the null hypothesis. From the likelihood ratio test of all the independent variables, a *p*-value of 0.0001 was obtained, indicating a statistically significant overall model, as shown in Table 4. The model was then tested to identify which of the independent variables have a significant effect on the dependent variable. The main effects were tested using one independent variable at a time versus the dependent variable. Variables with a significance value of less than 0.05 showed that they had an apparent effect.

Additionally, interactions between the variables were considered and tested to identify whether they had any significant effect on the duration. Interactions tested whether the product of two or more variables influenced the relationship between the independent and the dependent variables. The results of main effects and interactions of the presence of attractant, sheep personality type, and frequency on the duration are shown in the following subsections. The response variable alone showed no effect and, therefore, it was excluded from the results. Additionally, the interaction effects of variables on the duration were tested, e.g., interactions of sheep type*frequency on duration were explored because this yielded further information on which frequency bands play a role in the duration of the animals response based on the sheep personality type (i.e., bold vs. shy).

### 4.3. Exploring the Effects between the Independent Variables and the Dependent Variable

#### 4.3.1. Presence of Attractant vs. Stimulus Duration

**Figure 2:**
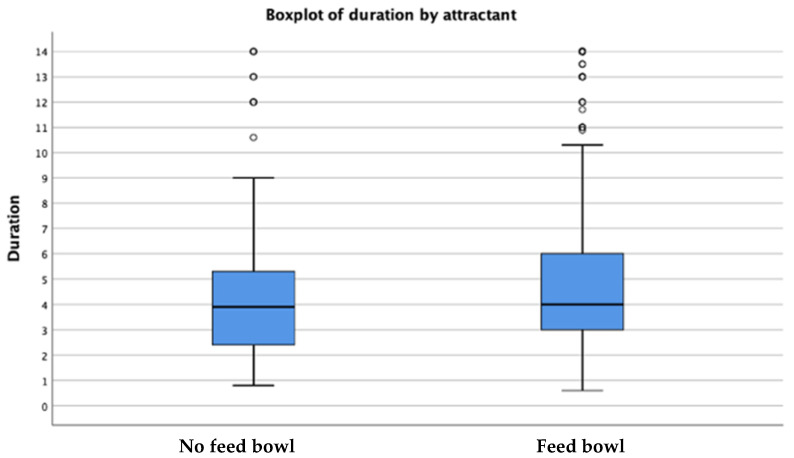
Time duration (in seconds) that animals need to respond to sound cues when an attractant is present/absent. The vertical lines indicate the range (minimum and maximum time in seconds), upper and lower quartiles with median (blue boxes), and outliers (represented by dots).

The average duration (seconds) of the animal response time when exposed to a sound is shown in Table 5. A relatively longer average response time of 4.60 s was observed when the area was provided with a feed bowl as compared to 3.19 s of the response time in the absence of the feed bowl. The average differences of these response times for the two categories (presence and absence of feed bowls) were observed to be statistically significant showing a faster average response time of 1.41 (4.60–3.19) s in the absence of the feed bowl (Figure 2 and Table 5). The boxplot in Figure 2 shows that the maximum time in which to respond when there was no attractant is approximately 1.4 s faster than when there was a feed bowl. The median time in both situations is 4 s, and the minimum time is little under 1 s. 

#### 4.3.2. Sheep Personality Type vs. Duration 

**Figure 3:**
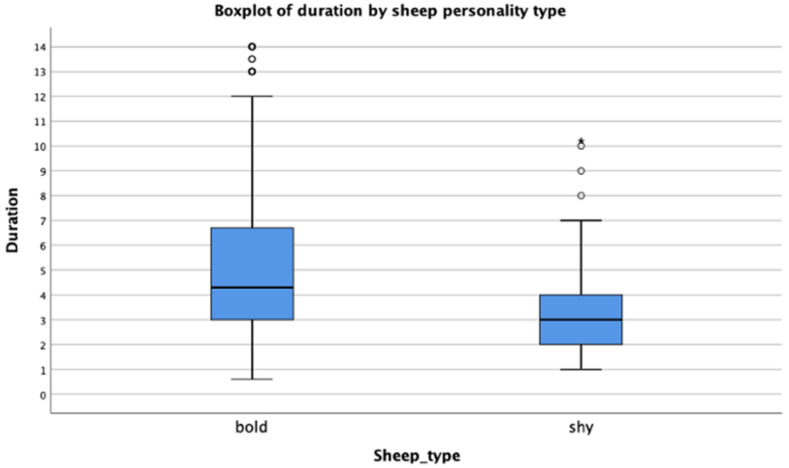
Time duration (in seconds) that animals need to respond to sound cues, based on sheep personality type. The vertical lines indicate the range (minimum and maximum time in seconds), upper and lower quartiles with median (blue boxes), and outliers (represented by dots).

As shown in Table 6, bold animals respond to sounds with a mean duration of 5.17 s, in contrast to shy animals with a mean duration of 3.05 s. Pairwise comparisons indicated statistical significance between sheep personality type and the sheep response time (*p*-value of 0.0001), having a mean difference of a 2.12 (5.17 s–3.05 s) s faster response than when the animal is considered shy. Figure 3 shows that shy animals need less time to respond to sounds compared to bold animals. The maximum time in which shy sheep responded was 7 s, while for bold animals this was 12 s. On the other hand, the minimum time for both personality types to respond was just under 1 s with a median of 4 s and 3 s for bold and shy sheep, respectively.

#### 4.3.3. Frequency vs. Duration

Results indicate no significant difference by means of duration between the four frequency bands. Table 7 shows that only the low frequency band (125–440 Hz) has a faster response with a mean of 3.12 s, in comparison with the remaining three (white noise, 10–14 kHz, and 15–17 kHz). A pairwise correlation analysis of each frequency band and their pairwise comparison using the Wald chi-squared test showed a *p*-value of 0.023. Further analysis of the frequency indicated a statistically significant relationship between white noise and the 125–440 Hz band with a mean difference in time to respond of 1.35 s. The frequency bands of 10–14 kHz and 15–17 kHz were found not to be significantly different from white noise. The overall estimate of the main effect between frequency and duration has a significant effect with a *p*–value of 0.033. 

### 4.4. Exploring the Interaction Effects of Sheep Type and Frequency on Duration 

Table 8 presents the model-estimated marginal mean, standard error, and confidence interval of the duration when considering interactions between sheep type and frequency category. From Table 8, it can be observed that that the mean duration ranges from a low of 2.39 s for shy sheep exposed to sounds from the 125 –440 Hz band, to a high of 6.07 s for bold sheep exposed to sounds from the 10 k–14 kHz frequency band. Levels of significance in the relationship between personality type and frequency band are discussed in the next section.

Table 8 represents statistically significant effects. The results in Table 8 indicate that the interaction effect of the combination of bold personality*frequency on duration is statistically significant between the frequency band 125–440 Hz vs. 10–14 kHz and 15–17 kHz, with *p* = 0.026 and *p* = 0.028, respectively. Bold animals react 1.99 s faster when sounds are emitted at the 10–14 kHz band compared to the 125–440 Hz band, and 1.94 s faster when 15–17 kHz sounds are emitted compared to those of the 125–440 Hz band. On the other hand, the pairwise comparison between frequency bands and shy personality sheep shows a significant difference with a *p*-value of 0.033 between sounds in the 125–440 Hz band vs. white noise, where in this situation, shy sheep react 1.81 s faster in the case of 125–440 Hz. There is an overall significance value of 0.0001 on the effect of the combination of sheep type*frequency band.

## 5. Discussion

In this research, a systematic analysis of the effects of audio stimuli and associated parameters as a means of influencing animal response, without the application of electric shocks, in a flock of Hebridean ewes was presented. Previous studies have shown that, without prior warning of an aversive cue such as the visual tape of an electric fence or the audio warning prior to the electrical stimulus, animals are at risk of becoming confused and may experience helplessness and hopelessness [31,32]. In the experiments performed in this work, associative learning using acoustic stimuli was used, starting benignly and then increasing in volume as punishment, in order to provide animals with the necessary warning to stop once the audio warning was heard right at the start.

The technology of virtual fencing systems based only on sound is not new; however, it has been mostly applied on cattle [21,33]. Studies of virtual fence systems for sheep solely based on sounds are limited and most of them have used a combination of auditory warning and electric stimuli to train the animals [31,34,35,36]. It has been reported that training animals using an electric stimulus could have a negative impact on their welfare [15,16], and this has led to their use on sheep being made illegal, as is the case in the UK [17,37].

### 5.1. Frequency Bands vs. Response vs. Sheep Personality Type

Overall, the animals reacted satisfactorily, with response levels over 88.48% for all four selected sounds. A total of 89.88% of the response times indicated that it is possible to monitor the animals’ location on the land they graze. The most desired response of the animals was to turn and walk away calmly, suggesting that the emitted sound does not cause any stress on the animal [31,35,36]. From the above results, the following observations may be drawn. White noise appears to be more alarming to animals than the rest of the frequency bands, since for 20.50% of the times, the recorded response was to turn and run away. On the other hand, when sounds in the higher frequency band of 15–17 kHz were emitted, the animals either turned and walked away with a rate of 89.77%, or stopped with a rate of 10.23%. Using this band, the animals did not run away and this indicates that this band was successful in managing animal behaviour while not causing unnecessary stress. Moreover, when sounds in the frequency band of 10–14 kHz were emitted, the animals turned and walked away 81.71% of the time, and turned and ran 4.88% of the times, and stopped with a rate of 13.23%. This band attained the second highest response with 89.13% of responses. The literature reports that sheep can hear best at 10 kHz [18], and this might be the reason for the high rate of occurrence of desired reactions. 

From the results section, it is suggested that all four sounds (i.e., white noise and selected frequency bands) could be used in the conceptual design of a virtual fencing system for shy animals. However, some sounds may be subject to habituation. The results suggest that, in a virtual fence system, three bands of 125–440 Hz, 10–14 kHz, and 15–17 kHz could be randomly played to achieve one of the desired behavioural reactions. It was observed that white noise caused more stress/irritation to the animals based on their reaction (i.e., turned and ran away from the area), and thus it may not be subject to habituation. Our suggestion is in agreement with Umstatter et al., i.e., the development of a smart virtual fence should trigger different sounds in a random pattern to avoid habituation, while white noise could be used as a last resort [33]. Based on these results, it could be concluded that the temperament of an animal plays an important role in terms of their behavioural response to the use of a virtual fence. Shy animals reacted as desired with a rate of 98.40% and additionally often did so when bold animals were wearing the collar, and reacted as desired. Therefore, it could be implied that audio cues were successful at restricting sheep in accessing a restricted area and that they have potential as a replacement tool to using electric stimuli in a VF system. The ability of the animals to learn a virtual fence system based on their temperament needs to be further investigated. Other studies have found no association between temperament and learning [31]. Sheep have excellent learning and memory abilities and can follow sophisticated rules including reversal learning [10,16,38,39] and response inhibition [40]. Furthermore, the time required for training sheep is markedly shorter than has been reported in primates, where training and testing typically takes many months [38]. In this study we demonstrated experimental evidence that that the response inhibition capability in sheep individually or in small flocks could be linked to control of the position via an acoustic stimulus to instruct animals to stop moving in one direction and take another away from a virtual boundary.

### 5.2. Main Effects and Interactions of Sheep Personality Type, Presence of Attractant, and Frequency on Duration

#### Attractant vs. Duration and Sheep Personality Type vs. Duration

From the results obtained while analysing the main effect of the attractant on duration needed for the animal to respond, it was confirmed that the mean difference of the estimations is statistically significant (*p* = 0.016). The animals reacted faster to the exposure of the sounds, when there is no feed bowl involved. This suggested that the sound somewhat irritated the animals and, thus, they reacted faster when there was no motivation/reward. In a real-world scenario, this could indicate that, if a virtual fence is used in a pasture, where taller and better grass is available in the restricted area, the animals may be willing to attempt to cross over more times. On the other hand, they could be easily manipulated with the emission of sounds if the restricted area is not as attractive to them. 

### 5.3. Bold vs. Shy Animals

Based on the results, the band of 125–440 Hz showed a dominant response for bold animals and needs further investigation. The same was observed with shy animals for the same frequency range. In this situation, low-frequency sounds caused a faster and more statistically significant response of approximately 2 s (*p* = 0.033) in comparison to white noise. The reaction to the low frequencies is interesting and it is worth investigating further with larger flocks and different animal breeds. This frequency band may be more alarming, or the observed behavioural responses may have been due to unknown factors related to the hearing sensitivity of sheep, which could be, e.g., associated with age [2].

## 6. Conclusions and Future Directions

The present study demonstrated a potential alternative to the use of electric shocks for sheep behavioural management with the use acoustic cues, thus promoting the design of future virtual fencing systems. Recent literature has also indicated that there is considerable evidence for such developments to be further explored in commercial applications. In the study, four frequency bands were identified, which favourably influenced behavioural responses, related to the spatial distribution of sheep, specifically, restricting access to a feed bowl or a specified area with an overall success rate of 89.88%. White noise introduced overt stress to the sheep, as the sheep turned and ran away from the area on 20.50% of the occasions. Animal personality (i.e., bold vs. shy) has a significant correlation with their reaction time, with shy animals having a faster reaction time to avoid continued emission of auditory stimuli. In addition, the presence of a feeding bowl decreases the reaction time of the sheep, so they react much more slowly to sounds. 

The evidence collected in the present study through two trials can be vital in understanding the potential for using acoustic stimuli in VF systems and restricting access to areas with taller and higher quality grass. The utilization of a VF system in such settings can be challenging as the animal persistence for feeding may overcome their fear of an acoustic signal for managing the spatial position. 

The results from this study are promising; however, the work conducted used a small group of animals. Lastly, it was observed that lower frequencies cause a significantly faster reaction, and further investigation with diverse experiments using a lower frequency band can be significantly useful for the validation of the VF systems. It is noted that, although the present study identified various useful correlations based on two trials, further investigation with larger flocks and other animal species is required for the validation of VF systems and their applicability for wider settings. Additionally, future work should examine the association between age and response to sound.

## Figures and Tables

**Table 1:** Frequency bands and number of repetitions in trial 1.

Sound	Total	No Response	Total Response	Turn + Walk Away	Turn + Run	Stop
White noise	52	0 (0.00%)	52 (100%)	22 (42.31%)	25 (48.08%)	5 (9.62%)
125–440 Hz	35	9 (25.71%)	26 (74.29%)	18 (69.23%)	5 (19.23%)	3 (11.54%)
1–5 kHz	19	10 (52.63%)	9 (47.37%)	3 (33.33%)	6 (66.67%)	0 (0.00%)
6–9 kHz	17	11 (64.71%)	6 (35.29%)	4 (66.67%)	2 (33.33%)	0 (0.00%)
10–14 kHz	19	4 (21.05%)	15 (78.95%)	10 (66.67%)	4 (26.67%)	1 (6.67%)
15–17 kHz	38	6 (15.79%)	32 (84.21%)	28 (87.50%)	0 (0.00%)	4 (12.50%)

**Table 2:** Frequency bands and number of repetitions in trial 2.

Sound	Total	No Response	Total Response	Turn + Walk Away	Turn + Run	Stop
White noise	207	20 (9.66%)	187 (90.34%)	139 (74.33%)	24 (12.83%)	24 (12.83%)
125–440 Hz	112	8 (7.14%)	104 (92.86%)	84 (80.77%)	5 (4.81%)	15 (14.42%)
10–14 kHz	73	6 (8.22%)	67 (91.78%)	57 (85.07%)	0 (0.00%)	10 (14.93%)
15–17 kHz	205	22 (10.73%)	183 (89.27%)	165 (90.16%)	0 (0.00%)	18 (9.84%)
Average	597	56 (9.38%)	541 (90.62%)	445 (82.26%)	29 (5.36%)	67 (12.38%)

**Table 3:** Dependent variable statistics.

		Minimum	Maximum	Mean	Std. Deviation
Dependent Variable	Duration (in seconds)	1	14	4.92	3.316

**Table 4:** Results from the overall model.

Likelihood Ratio Chi-Square	Degrees of Freedom	Significance Level
132.635	22	0.000

**Table 5:** Estimates of duration (in seconds) in the presence/absence of attractant.

Attractant	Mean	Std. Error	Sig.	95% Wald Confidence Interval	
				Lower	Upper
No feed bowl	3.19	0.505	0.016	2.34	4.35
Feed bowl	4.60	0.342	0.016	3.97	5.32

**Table 6:** Estimates of duration (in seconds) by personality type.

Sheep_Type	Mean	Std. Error	Sig.	95% Wald Confidence Interval	
				Lower	Upper
bold	5.17	0.365	0.000	4.50	5.94
shy	3.05	0.374	0.000	2.40	3.88

**Table 7:** Estimates of duration (in seconds) by frequency band.

Frequency	Mean	Std. Error	Sig.	95% Wald Confidence Interval	
				Lower	Upper
white noise	4.48	0.623	0.023	3.41	5.88
125–440 Hz	3.12	0.346	0.023	2.52	3.88
10–14 kHz	4.25	0.497	>0.05	3.38	5.35
15–17 kHz	4.19	0.630	>0.05	3.12	5.62

**Table 8:** Estimates of duration (in seconds) by the combination of sheep type*frequency.

Sheep Type	Frequency	Mean	Std. Error	Sig.	95% Wald Confidence Interval	
					Lower	Upper
bold	white noise	4.77	0.497	>0.05	3.89	5.85
	125–440 Hz	4.09	0.389	0.026	3.39	4.92
	10–14 kHz	6.07	0.801	0.026	4.69	7.86
	15–17 kHz	6.03	0.893	0.028	4.51	8.06
shy	white noise	4.20	0.904	0.033	2.76	6.41
	125–440 Hz	2.39	0.413	0.033	1.70	3.35
	10–14 kHz	2.98	0.573	>0.05	2.04	4.35
	15–17 kHz	2.91	0.532	>0.05	2.03	4.16

## Data Availability

Not applicable.
